# The Vital Statistics and Sanitary Condition of Memphis, Tennessee, an Anniversary Address, Delivered by Appointment, before the Memphis Medical Society, on the 5th of February, 1852

**Published:** 1852-08

**Authors:** 


### BIBLIOGRAPHICAL NOTICES.

---

ART. V.—*The Vital Statistics and Sanitary Condition of Memphis, Tennessee, an Anniversary Address, delivered by appointment, before the Memphis Medical Society, on the 5th of February,* 1852. By GEO. R. GRANT, M. D.

Dr. Grant, when he commenced the investigation, the results of which are embodied in this pamphlet, had the conviction, "that Memphis is one of the healthiest places on the Mississippi river." He believed that a comparison of the sanitary condition of that city with that of "other places in the great valley, and also with other cities in the Union, would conclusively establish the truth of this opinion." He says, he entered with alacrity on the self-imposed task of disproving by figures the charge, that Memphis is a sickly place, but on inquiry he was forced to conclude that, in this instance, common rumor had too much truth in it. We make a few extracts from his interesting address.

The *white* population of Memphis, according to the census of 1850, was 6,369. Of this number, 3,587 were males, and 2,782 were females. There were also, 44 free *colored* males, and 65 free *colored* females. As the deaths among these 109 free persons of color were not kept separate, but included in the returns among the whites as free persons, their numbers must be added to the whites, in order to arrive at the per centage of mortality among the persons, white and colored, composing our white population. Among this white and colored free population, the returns give 238 deaths; a per centage of 3.67, or 36 in every thousand. From the same authority we learn, that the slave population numbered 2,362; of whom 116 had died; making the per centage of deaths among this class 4.91, or 49 out of every thousand. The average mortality of these two classes together, gives the astonishing result of a fraction over 4 per cent., or 1 death in every 25 living!

The returns of the City and County being kept separate, made it, comparatively, an easy task to examine the *vital statistics* of our immediate neighborhood, and therefrom collect materials wherewith to compare our sanitary condition with that of the people by whom we are surrounded. These statistics show a population in the county, exclusive of the city, of 10,317 free persons, and 11,998 slaves; making a total of 22,315. The deaths among the free were 190, being 1.84 per cent; and among the slaves, 279, giving 2.32 per cent. The average ratio of mortality among our neighbors turn, out to be only 2. 1-10 per cent of the entire population, or one in every 47 living. Here we have proved to us, by unmistakeable data, that whilst one in every 25 had died in town, the people who are separated from us by a mere compass line, have lost but *one*, by death, out of every 47 living. This difference in the mortality of the city over the county, of very nearly *two to one*, is well calculated to excite the surprise of the statist, and the sympathy of the philanthropist; while it offers to the medical philosopher an open and interesting field for etiological investigation.

If the foregoing statements are calculated to astonish us, what will we say when we compare our sanitary condition with that of New Orleans, as shown from the late census returns of that city, the vital statistics of which have been carefully collected by Dr. Barton, and published in the last volume of the "Southern Medical Reports." According to these returns, we find the mortality in New Orleans and Lafayette, *exclusive* of cholera, to be a fraction less than 2 per cent., (1.96) and *including* cholera, it is less than 2½ per cent; being 2.44. This shows a lower rate of mortality, by more than one and a palf per cent. in New Orleans than occurred in Memphis during the same period of time; the facts being collected in a similar manner; ours, by one of our own citizens, who is in every respect well qualified for the proper performance of the duties the office enjoined.

It would be a fruitless labor to extend our inquiries to other cities of the Union, with the hope of finding in the census returns of 1850, any mortuary statistics at all approximating to the figures exhibited by the recorded deaths among our population, for the period embraced therein. Every comparison of the sort would only be to our disadvantage.

Dr. Grant finds that the mortality in the Memphis Hospital is also greater than in other similar institutions in our country.

The total number of admissions into the Memphis Charity Hospital for the year ending on the 31st of December last, was 474. The books show that 116 of these had died; making a mortality of 24.47 per cent; wanting a fraction only of being one out of every four, or very nearly 24½ out of every hundred! That this is a terrific mortality, will become quite evident by comparing it with the annual mortality witnessed in other Hospitals—some of them in localities confessedly insalubrious.

From estimates made from twelve years' observations by Dr. Playfair, in the Hospitals of Liverpool and Manchester, the mortality was 3.57 per cent., or one in every 28. From a statement published in the January number of the "London Lancet" for the present year, we learn that there had been admitted into all the Hospitals of Paris, during the year 1850, 84,044 patients. Of these, 6,855 had died; making an average mortality of 8.15 per cent., or one in every 12½.

In the Marine Hospital at Louisville, Kentucky, as we are informed by Dr. Rogers, in the December number of the Western Medical and Surgical Journal of last year, the mortality was a small fraction over 7 per cent., or one in every 14.

According to a statement published by Dr. McKelvey, the Surgeon to the United States Marine Hospital at New Orleans, out of 1,116 admissions in the year 1849, there occurred but 64 deaths; making a mortality of only 5.73 per cent., or one in every 17. In the Charity Hospital,—

that immense lazaretto of New Orleans,—the admissions in 1850 numbered 18,676 patients, of whom 1,884 died—being a mortality of 10 per cent., or one in every 10.

From these comparative hospital statistics, it is but too evident that the mortality at our Hospital is more than twice as large as that of the Charity Hospital at New Orleans, which has always been considered, in this quarter, as the great Southern receptacle of the sick and the dying!

As any farther inquiry would serve only to increase the melancholy reflections which naturally intrude, when we compare the picture presented by our Hospital returns, with that of similar institutions in this or other countries, we will leave this branch of our investigation, and proceed to give the returns collected from the records kept by the Secretary of the Board of Health.

These momentous facts established by the researches of Dr. Grant ought to attract the attention of the municipal authorities of Memphis. There can be no doubt that there is a needless mortality in all great cities—a frightful waste of human life which a wise provision might prevent. We gave some details respecting the mortality of Boston in a late number. It appears from a statistical report by one of the municipal officers of New York, recently made public, that the deaths in that city during the year 1851 reached the enormous aggregate of twenty thousand and twenty-four, an increase of five thousand and forty-six, or nearly thirty per cent. over the number in 1850. The increased mortality took place chiefly among children; the increase in adults being 1,264, and in children, 3,782. The number of deaths from consumption during the year was exceedingly large, but the greatest increase was in those classes of disease which are propagated by contagion, and have their causes in the impurity of the atmosphere. The deaths by typhus fever increased from 396 to 977; by scarlet fever from 311 to 627; by dysentery from 792 to 1,113; by diarrhea from 473 to 743; and by convulsions from 1,288 to 1,792.

Compared with London, the mortality in New York is as one in thirty-one to one in forty-eight. A writer in one of the papers of that city, says there is something startling in these facts, and adds:

If there is any truth in official figures, the fact is incontrovertible that, in spite of increased wealth, improved medical skill, and the introduction and almost universal use of that invaluable blessing, the Croton water, the active causes of disease and death are increasing among us; and that the actual duration of life is yearly diminishing.

